# The effects of a bioavailable curcumin formulation on Alzheimer's disease pathologies: A potential risk for neuroinflammation

**DOI:** 10.1002/ibra.12187

**Published:** 2024-12-11

**Authors:** Shaun Cade, Clive Prestidge, Xin‐Fu Zhou, Larisa Bobrovskaya

**Affiliations:** ^1^ Health and Biomedical Innovation, Clinical and Health Sciences University of South Australia Adelaide South Australia Australia; ^2^ Center for Pharmaceutical Innovation, Clinical and Health Sciences University of South Australia Adelaide South Australia Australia

**Keywords:** Alzheimer's disease, amyloid pathology, curcumin, neurodegeneration

## Abstract

Alzheimer's disease (AD) is a common cause of dementia characterized by the presence of two proteinaceous deposits in the brain. These pathologies may be a consequence of complex interactions between neurons and glia before the onset of cognitive impairments. Curcumin, a bioactive compound found in turmeric, is a promising candidate for AD because it alleviates neuropathologies in mouse models of the disease. Although its clinical efficacy has been hindered by low oral bioavailability, the development of new formulations may overcome this limitation. The purpose of this study was to determine the effects of a bioavailable curcumin formulation in a mouse model of AD. The formulation was administered to mice in drinking water after encapsulation into micelles using a previously validated method. A neuropathological assessment was performed to determine if it slows or alters the course of the disease. Cognitive performance was not included because it had already been assessed by a previous study. The bioavailable curcumin formulation was unable to alter the size or number of amyloid plaques in a transgenic mouse model. In addition, mechanisms that regulate amyloid beta production were unchanged, suggesting that the disease had not been altered. The number of reactive astrocytes in the hippocampus and dentate gyrus was not altered by curcumin. However, protein levels of glial fibrillary acidic protein were increased overall in the brain, suggesting that it may have aggravated neuroinflammation. Therefore, a higher dosage, despite its enhanced oral bioavailability, may have a potential risk for neuroinflammation.

## Graphical abstract

Curcumin has shown promise as an alternative therapeutic for Alzheimer's disease (AD) in lab‐based studies. However, its effectiveness in humans with the disease has been limited due to low oral bioavailability. Formulations with improved oral bioavailability have been developed to overcome this limitation, and further testing of these formulations may increase their use. This study performed a neuropathological assessment in AD mice treated with a bioavailable curcumin formulation. The main outcome measures were amyloid plaque deposition, mechanisms that regulate amyloid‐beta production, and astrocyte reactivity. Unfortunately, most outcome measures were unchanged, except for a reduction in total protein levels of c‐Jun‐N‐terminal kinase. A higher dosage may have been more efficacious.

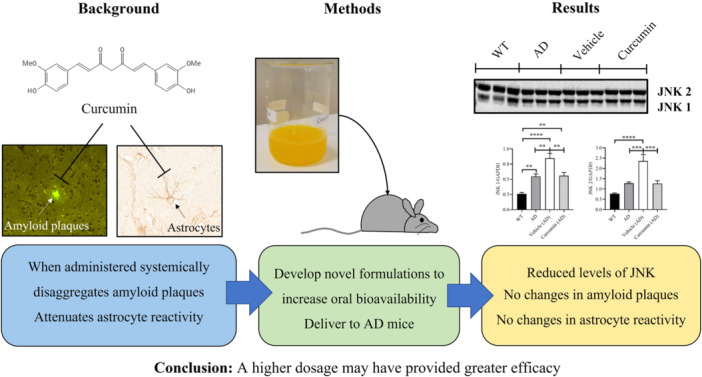

## 1 INTRODUCTION

Alzheimer's disease (AD) is a common cause of dementia that will likely become more burdensome due to population aging.^1^ The number of people with dementia worldwide is expected to reach 152.8 million by 2050, more than double of those affected in 2019.^1^ This forecast necessitates present‐day strategies, such as the development of more effective treatments, to mitigate its future effects.^2^ The most common cause of dementia is AD, a neurodegenerative condition defined by the presence of two proteinaceous deposits in the brain.^3^ These deposits include amyloid plaques composed of the amyloid beta (Aβ) peptide and neurofibrillary tangles (NFTs) composed of hyperphosphorylated tau.^4^ Evidence suggests that the abnormal aggregation of Aβ precipitates the pathological processes of AD years before the onset of cognitive impairment.^5^ Aβ oligomers, intermediates between monomers and fibrils, stimulate hyperphosphorylation of the amyloid precursor protein (APP) and tau.^6^, ^7^ This abnormal biochemical state in neurons may exacerbate Aβ production and facilitate NFT formation through the neurotrophin receptor, p75NTR.^8^, ^9^, ^10^ However, the pathological mechanisms of AD are not limited to neurons as excessive reactions by neuroglia also play a role.^5^ Astrocytes surrounding amyloid plaques become reactive, whereby they extend out their processes and secrete chemokine ligands that attract microglia to the site.^11^ This response creates a toxic microenvironment that worsens as the disease progresses and relates to symptom duration.^12^ To slow or halt AD, therefore, it may be necessary to target multiple pathological mechanisms.

Curcumin is a natural plant‐based product that may alter the course of AD with improvements in its oral bioavailability.^13^, ^14^ Standard curcumin has been shown to target multiple AD‐related pathologies in transgenic mice. It reduces the number and size of amyloid plaques, attenuates astrocyte reactivity, and protects against synapse loss, suggesting that it may have disease‐modifying potential.^15^, ^16^, ^17^ However, the promising effects of curcumin are yet to translate into humans as it has been ineffective in clinical trials.^18^ Even with improvements in its oral bioavailability, the major limitation, biomarkers of disease pathology and cognitive function are unaltered.^19^ Nevertheless, several curcumin formulations with enhanced solubility and stability have since been developed.^14^, ^20^, ^21^, ^22^ Some of these formulations alleviate pathologies in rodent models of AD more effectively than standard curcumin, suggesting that they could be effective in humans.^13^, ^20^ Further development and testing of novel formulations may provide additional support for the use of curcumin as a therapy for AD.

The curcumin formulation used in this study was previously optimized to improve its solubility and stability for oral administration.^21^ Its effects on cognitive performance in AD transgenic mice and pathologies in a mouse model of Parkinson's disease have previously been assessed.^13^, ^23^ However, its effects on neuropathologies in AD transgenic mice have not been evaluated, so its preclinical evaluation remains incomplete. The purpose of this study was to perform a neuropathological assessment in AD transgenic mice treated with the formulation. The mice used to model AD were of the double transgenic APPswe/PS1dE9 genotype, which has mutations linked to familial AD.^24^ These mice have been used extensively to test the effects of novel compounds and formulations and were suitable for use in this study.^23^, ^25^, ^26^ Amyloid plaques were detected using Congo red according to a method that increases its specificity,^27^ and imaged with fluorescence microscopy. Although fluorescence increases the detection of plaques, it can still give false‐positive results,^28^ so immunohistochemistry was also performed using an antibody specific to Aβ.^29^ Mechanisms indirectly related to Aβ production were measured to determine how curcumin produces anti‐amyloidogenic effects. Neuroinflammation, as reflected by astrocyte reactivity, was also assessed in the hippocampus and whole brain homogenate.

## 2 MATERIALS AND METHODS

### 2.1 Drugs, chemicals, and reagents

Curcumin powder (≥ 60% curcumin) was obtained from Chem‐Supply (Glentham Life Sciences, AUS, cas #458‐37‐7; batch #341QFC) and stored at −20°C. Soluplus was obtained from BASF (lot #85937736W0) and stored at room temperature. Congo red powder was obtained from Sigma Aldrich (cat #C‐6277). The microBCA protein assay kit was obtained from Thermo Scientific (US, cat #23235).

### 2.2 Curcumin preparation

Curcumin was encapsulated by Soluplus using a method previously developed for optimization of its solubility and stability.^21^ Briefly, curcumin was dissolved in absolute ethanol; then, Soluplus was gradually added while stirring until the final ratio of curcumin to drug carrier was 1:10. Ethanol was removed by rotary evaporation in a water bath (25°C) while rotating at low speed. Once a crystalline structure appeared, the flask was removed, covered in parafilm, and allowed to dry for 48 h. The crystalline product was then scrapped with a metal utensil and ground into a fine powder using a mortar and pestle. The powder was placed inside a falcon tube, wrapped in foil then stored at −20°C.

### 2.3 Oral delivery

Encapsulated curcumin was dissolved in water (pH 4.0) at 2.0 mg/mL in a glass beaker wrapped in foil. The concentration was calculated from daily water consumption and aimed to deliver a dosage of 24 mg/kg/day. The solution was stirred at medium speed for 24–48 h or until dissolved. The formulation was then poured into drink bottles without filtration. Bottles were wrapped in foil to prevent exposure of the solution to light and changed weekly.

To prepare the vehicle solution, Soluplus was dissolved in water at the same concentration as the curcumin preparation. The solution was stirred at medium speed for about 1 h and then poured directly into fresh drinking bottles. Bottles were wrapped in foil and changed weekly.

### 2.4 Animals

Male and female APPswe/PS1dE9 mice on a C57/BL6J background were used to model AD (AD mice). These mice have a chimeric APP transgene with a human sequence in the Aβ region and mutations linked to a Swedish variant of familial AD.^24^ They also express mutant presenilin‐1 (PS1), a component of the γ‐secretase complex, and develop amyloid plaques by 6 months of age.^24^ Age‐matched male and female C57/BL6J mice (WT mice) were used as controls. In total, 32 mice were used in the study. All mice were bred and housed in the core animal facility of the University of South Australia. Mice were housed in filtered, individually ventilated cages (IVC's) in a room maintained at 20.5–23.5°C with a 12‐h light/dark cycle. They were fed with autoclaved “Standard Rat and Mouse Chow,” (Specialty Feeds, AUS) and supplied with acidified drinking water (pH 4.0). All experiments and procedures were performed in accordance with the approved animal ethics protocol. All animal procedures in this study were approved by the University Animal Ethics Committee, University of South Australia (U45/19, approval date November 8, 2019).

### 2.5 Experimental design

A total of four groups of mice were used for this study including baseline controls consisting of WT and AD mice aged 9 months (Table 1). The therapeutic groups consisted of AD mice treated with curcumin (24 mg/kg/day) and another group treated with vehicle (264 mg/kg/day) (Table 1). The number of mice per group was derived from power calculations based on previously published studies.^23^, ^25^ In these studies, the outcome measure used in these calculations was cognitive function as no neuropathological assessment had been performed for curcumin. To achieve 80% statistical power, seven mice per group were needed. However, an extra mouse was added per group (*n* = 8) due to higher rates of seizures among AD mice.^30^ All groups consisted of an equal proportion of males and females.

**Table 1:** Experimental groups including drug treatments and doses for the therapeutic study.

Group	Genotype	Age (months)^a^	Treatment	Dose	Number (*n*)
Ⅰ	C57/BL6J	9	Baseline control	N/A	8
Ⅱ	APP/PS1	9	Baseline control	N/A	8
Ⅲ	APP/PS1	14	Vehicle: Soluplus	264 mg/kg/day	8
Ⅳ	APP/PS1	14	Curcumin	24 mg/kg/day^b^	8

^a^ Age at the time of tissue collection.

^b^ Curcumin content only, not including Soluplus.

**Figure 1:**
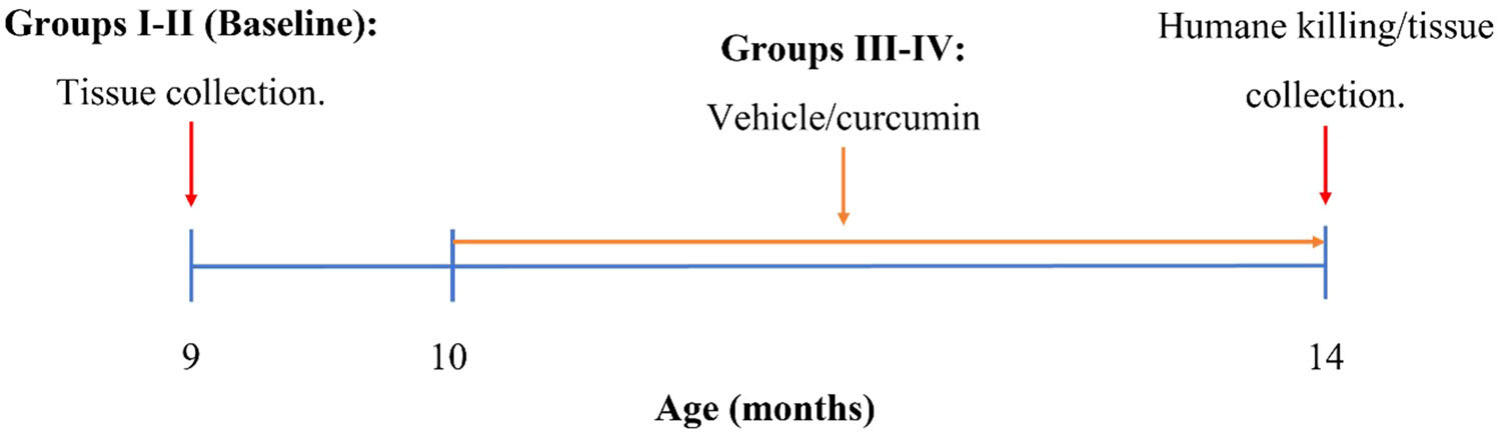
Experimental timeline showing the age of tissue collection for the baseline controls and duration of treatment for vehicle and curcumin groups. [Color figure can be viewed at wileyonlinelibrary.com]

Tissues were collected from WT and AD mice aged 9 months (baseline) for comparison with therapeutic groups (Figure 1). Curcumin and vehicle were administered to AD mice aged 10 months for 4 months before humane killing and tissue collection (Figure 1).

### 2.6 Humane killing and tissue collection

Mice were humanely killed using CO_2_ for 5 min then transcardially perfused with ice‐cold 1x phosphate‐buffered saline (PBS). The whole brain, including the cerebellum and brain stem, was then removed and cut longitudinally with a scalpel. The right hemisphere was snap‐frozen on dry ice and the left was immersed in room temperature 10% formalin/1x PBS. Snap‐frozen tissues were later transferred to −80°C storage while formalin‐fixed tissues were placed in 4°C storage for 24 h before transfer to cold 1x PBS + 0.02% sodium azide.

### 2.7 Histology

#### 2.7.1 Processing and embedding

Before processing and embedding, the left brain hemisphere was sectioned using a brain matrix (Zivic instruments, cat #5325). The hemisphere was placed inside a mold of the mouse brain dorsal side up and then cuts were made at the levels of the cerebellum and striatum. The cerebellum and pre‐frontal cortex were discarded while the middle section was secured inside a tissue processing/embedding cassette (Simport, Canada, cat #M490). Cassettes were re‐immersed in 1x PBS + 0.02% sodium azide and kept in cold storage (4°C) until processing.

Plastic cassettes containing tissues were immersed in 70% ethanol and then transferred to an automated tissue processor (Leica ASP300 S). Tissues were dehydrated overnight with ascending concentrations of ethanol (70%, 80%, 90%, and 100%) followed by clearance with xylene before embedding in paraffin. The following day, the cassettes were transferred to hot wax and embedded in molds.

#### 2.7.2 Sectioning

Tissue blocks were precooled for 30 min at −10°C before sectioning in a rostral‐caudal direction until the hippocampus emerged. Sections from this region and regions further caudal were collected. Four‐micrometer sections were expanded on a water bath set at 45°C before mounting onto slides (Ultra Plus, Thermo Scientific) in triplicate. The wax was then melted in an oven for about 2 h.

#### 2.7.3 Congo red

The working solution was prepared by dissolving 1 g of Congo red powder in 200 mL of alkaline alcohol (0.01% sodium hydroxide in 50% ethanol), stirring overnight, and then filtering the following day. Tissues were dewaxed in xylene for 2 min (repeated three times) followed by 100% ethanol for 2 min (repeated twice). They were then hydrated in 90% ethanol for 2 min, followed by 70% ethanol for 2 min, and washed in running milli‐Q water for 2 min. Tissues were then immersed in the working Congo red solution for 20 min. To remove the background, slides were slowly dipped in milli‐Q water several times before dipping quickly into alkaline alcohol (0.01% NaOH in 50% ethanol) 10 times. Slides were then rinsed under running tap water for 2 min. Tissues were serially dehydrated using ascending concentrations of ethanol (70%, 80%, and 90%) for 3 min per wash. This was followed by immersion in 100% ethanol for 3 min (repeated twice) and xylene for 3 min (repeated twice). Finally, coverslips were attached with DPX mounting medium.

#### 2.7.4 6E10 immunohistochemistry

Tissues were dewaxed in xylene for 5 min three times, and in 100% ethanol for 5 min three times, before hydration in 95% ethanol and in 70% ethanol for 3 min, respectively, followed by washing with milli‐Q water for 1 min three times. Slides were then rinsed under running reverse osmosis (RO) water for 2 min. For antigen retrieval, slides were immersed in 70% formic acid for 20 min at room temperature. They were then washed in 1x PBS (3 min) twice before being placed in a slide tray with RO water. For blocking, tissues were incubated in 1% normal horse serum (NHS)/1x PBS for 20 min. The solution was removed by gently tapping the slides. A dilution of the primary antibody (Mouse, αAβ_1–16_ Alexa Fluor488, COVANCE, cat #SIG‐39347, clone 6E10, 1:500) was prepared with 1% NHS/1x PBST (1x PBS + 0.3% Triton‐X). Tissues were incubated with the primary antibody overnight at room temperature in the dark. Slides were then immersed in 1x PBS (3 min) three times before being washed under running RO water for 2 min. They were then serially dehydrated in ascending concentrations of ethanol (70%, 80%, 90%, and 95%) (3 min each) followed by 100% ethanol (5 min) twice. Finally, tissues were cleared in xylene for 5 min twice before attaching a coverslip with DPX mountant.

#### 2.7.5 Glial fibrillary acidic protein (GFAP) immunohistochemistry

Tissues were dewaxed in xylene and 100% ethanol for 5 min twice, respectively, before hydration in ethanol solutions (95%, 90%, 80%, and 70%) (3 min each). To block endogenous peroxidases, slides were immersed in 50% ethanol/1x PBS with 0.3% H_2_0_2_ for 30 min. They were then washed in 1x PBS (5 min) twice. For antigen retrieval, slides were immersed in citrate retrieval buffer (10.5 g of citric acid per 5 L of milli‐Q water, pH 6.0) and heated in a microwave until boiling. After cooling, they were heated again for 10 min on medium before washing in 1x PBS (5 min) twice. To reduce nonspecific binding, tissues were incubated in 3% NHS/1x PBS for 30 min, then the solution was tapped from the slides. A dilution of the primary antibody (Sheep, αGFAP, homemade, 1:1000) was prepared using 1% NHS/PBST. Tissues were incubated with the dilution overnight at room temperature.

After overnight incubation, tissues were washed in 1x PBS for 5 min twice. A dilution of a biotinylated secondary antibody (Donkey, αSheep‐biotin, Homemade, 1:500) was prepared using 1% NHS/PBST. Tissues were incubated with the dilution for 1 h and then washed in 1x PBS (5 min) twice. A working streptavidin solution was prepared by diluting streptavidin‐horse radish peroxidase (HRP) (0.1 mg/mL) at 1:500 using 1% NHS/PBST. Tissues were incubated with the dilution for 1 h and then washed in 1x PBS (5 min) twice. A working 3,3’‐Diaminobenzidine (DAB) solution was prepared by adding 250 μL of 1% DAB to 250 μL of H_2_0_2_ (0.6%) in 5 mL of 1x PBS. Tissues were incubated with DAB for 7 min before dipping in milli‐Q water to stop the reaction. They were then washed in running RO water for 2 min before dehydration in ethanol solutions (70%, 80%, 90%, and 95%) (3 min each). Finally, tissues were cleared in 100% ethanol (5 min) twice followed by xylene (5 min) twice before attaching a coverslip with DPX mounting medium.

### 2.8 Western blot analysis

#### 2.8.1 Tissue homogenization

Brain hemispheres were first pulverized into a fine powder using liquid nitrogen before tissue lysis. Powdered brain tissue was then weighed (*n* < 30 mg) into precooled screw cap homogenization tubes (2 mL) with two glass beads. Ice‐cold RIPA buffer (50 mM Tris‐Cl pH 7.5, 150 mM NaCl, 1 mM ethylenediaminetetraacetic acid (EDTA) with pH 7.5, 0.5% Triton X, 0.5% sodium deoxycholate, 0.1% sodium dodecyl sulfate (SDS) + 1 protease inhibitor tablet, (ROCHE, Germany, cat #11 836 170 001) was then added in a 1:20 ratio (mg tissue: µL of buffer). Tubes were run through one cycle of homogenization in a tissue lysis machine (Precellys 24, Bertin instruments, France), with three pulses of 20 s each. The tissue lysate was transferred to pre‐cooled Eppendorf tubes (1.5 mL) and centrifuged at 13,600 rpm for 30 min at 4°C. The supernatant was collected and stored at −80°C.

The microBCA protein assay was used to determine protein concentrations in the samples. Briefly, the homogenate was diluted 1:200 with milli‐Q water and loaded in duplicate (100 µL/well) onto a 96‐well plate. Bovine serum albumin (BSA) standards (1.25–80 µg/mL) were included along with a blank control (milli‐Q water). The working reagent was prepared by diluting reagents A, B, and C (supplied by the manufacturer) in a 25:24:1 ratio, respectively. One hundred microliters of working reagent was added to each well, then the plate was gently mixed before incubation at 37°C for 2 h. Absorbance was measured at 560 nm using a Victor 3, multilabel plate reader (PerkinElmer). Absorbance readings of the standards were used to interpolate theoretical protein concentrations in the samples.

#### 2.8.2 Gel electrophoresis

Brain homogenate was diluted to 1.5 µg/µL using sample buffer (60 mM Tris pH 6.8, 40% glycerol, 2% SDS, 0.1% bromophenol blue, 375 mM dithiothreitol (DTT) (25%), and milli‐Q water. Samples were then briefly centrifuged and vortexed before boiling at 100°C for 10 min to denature proteins. Proteins in sample dilutions were separated based on molecular weight using electrophoresis. A separating gel was prepared (10% acrylamide, 25% separating buffer, 0.1% SDS, 7.5% Glycerol in milli‐Q water + 25 μL tetramethylethylenediamine [TEMED], and 125 μL 10% ammonium persulphate [APS] per 30 mL) and then poured into preassembled double‐wide glass plates (C.B.S. scientific). After setting, a separating solution (181.6 g of Tris per 500 mL of milli‐Q water pH 8.8 + 7.5 µL TEMED and 150 µL 10% APS per 10 mL) was prepared and poured on top before inserting a comb. The gel assembly was then placed in a separating apparatus (C.B.S. scientific) with 1x lower buffer at the bottom and 1x upper buffer at the top. A molecular weight marker (BIORAD, AUS, cat #1610374) was then loaded along with sample dilutions, and the separation ran at 120 V for 2 h or until the dye reached the buffer.

Following separation, proteins were transferred onto a nitrocellulose blotting membrane. The separating gel, sponges, and nitrocellulose membrane (0.45 µm, Amersham, GE Healthcare, cat #10600002) were first presoaked in 1x transfer buffer. They were then assembled into a cassette and placed in a transfer apparatus (C.B.S. scientific). The transfer was run overnight at 0.1 A. The following morning, the membrane was air‐dried for 1 h before washing in Ponceau S (0.5% Ponceau S/1% acetic acid) followed by RO water to visualize the bands. Ponceau S was removed by washing with TBST (TBS + 0.08% Tween 20) (5 min) with shaking three times. To block nonspecific binding, the membrane was washed with 5% milk/TBST for 1 h shaking. The membrane was then washed three times with TBST for 5 min. For blotting, the membrane was incubated with a primary antibody (Table 2) overnight at 4°C shaking. All primary antibodies were diluted in 5% BSA/TBST with 0.05% sodium azide, except GFAP, β‐actin, and GAPDH, which were diluted in 5% milk/TBST with 0.05% sodium azide. After overnight incubation, the membrane was washed with TBST (5 min) three times. An IR‐Dye conjugated secondary antibody (Donkey, αRabbit, LI‐COR, cat #926‐68073; Donkey, αGoat, LI‐COR, cat #926‐32214; Goat, αMouse, LI‐COR, cat #926‐32210) was then diluted 1:20,000 in TBST. The membrane was incubated with the appropriate secondary antibody for 1 h in the dark, shaking. The membrane was then washed with TBST (5 min) three times followed by milli‐Q water (5 min) shaking at room temperature. Blots were scanned using the Odyssey Clx infrared imaging system (LI‐COR, US) and quantified using Image Studio Lite, version 5.2.

**Table 2:** Primary antibodies used in western blot analysis experiments.

Antibody	Animal	Company	Cat #	Working dilution
Human sAPPα	Mouse	Home‐made	N/A	1:1200
pAPP (T668)	Rabbit	Cell Signaling	3823S	1:1000
APP C‐terminal	Rabbit	Calbiochem	171610	1:1000
pBACE1 (S498)	Rabbit	Thermo Scientific	PA512549	1:1000
BACE (D10E5)	Rabbit	Cell Signaling	5606P	1:500
GFAP	Sheep	Homemade	N/A	1:2000#
pJNK (Thr183/Tyr185)	Rabbit	Cell Signaling	4668	1:500
JNK	Rabbit	Cell Signaling	9252	1:1000
p75NTR	Rabbit	Homemade	N/A	1:1000
Sortilin	Rabbit	Osenses	OSSO00011W	1:5000
β‐actin	Mouse	Homemade	N/A	1:20,000
GAPDH	Sheep	Homemade	N/A	1:5000

### 2.9 Quantification of amyloid plaques

Tissues stained with Congo red and 6E10 immunohistochemistry were imaged using a fluorescent microscope, Olympus (CX40). Images were captured in TIFF file format and saved onto a USB then uploaded into ImageJ (National Institute of Health, US).^31^ The software was calibrated using the scale bar in the image, then the image was converted to greyscale (8‐bit). The threshold was then adjusted, the image converted to binary, and watershed applied. Measurements were set to record the number of objects within the outlined area, the %area fraction (plaque area/total area), and the total object area. All images from each brain region were measured in a single session and the data were exported as an Excel spreadsheet. In Excel, the number of objects was divided by the area of the brain region (amyloid plaques/mm^2^). Triplicate measurements were then averaged to generate a single number for each mouse. A total of four mice per treatment group were included in the final statistical analysis.

### 2.10 Quantification of GFAP‐positive astrocytes

Whole slides were imaged using an automated microscope, nanozoomer (Hamamatsu, Japan). Images were then analyzed with NDP View 2 software (Hamamatsu, Japan). The dentate gyrus and hippocampus were identified and outlined using Allen's online mouse brain atlas. GFAP‐positive astrocytes were counted manually and then divided by the area of each brain region (GFAP‐positive astrocytes/mm^2^). At least two tissues from each mouse were analyzed and averaged. A total of four mice per group were included in the final statistical analysis, except the vehicle group, which consisted of three mice.

### 2.11 Statistical analysis

All statistical analyses were performed using GraphPad Prism version 8.0. Data were expressed as mean ± standard error of mean (SEM). Before a test was selected, the distribution of the data were determined using normality and lognormality tests along with a Quantile–Quantile plot. For normal data, one‐way analysis of variance (ANOVA) followed by Tukey's multiple comparisons was used. For non‐normal data, Kruskal–Wallis followed by Dunn's multiple comparisons was used. *p* < 0.05 was considered statistically significant.

## 3 RESULTS

### 3.1 Curcumin had no effect on the number or size of amyloid plaques

**Figure 2:**
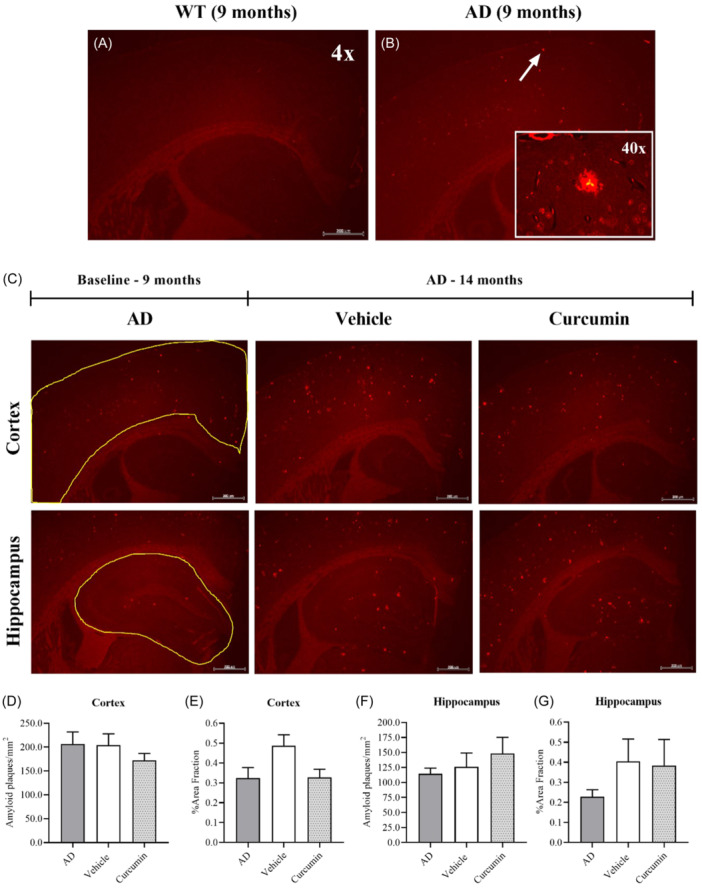
Detecting amyloid plaques in the cortex and hippocampus of AD mice with Congo red. (A, B) Brain sections from AD mice aged 9 months showed fluorescent particles (inset image) throughout the cortex when visualized under fluorescence microscopy. The particles were absent in WT mice of the same age, confirming that they were amyloid plaques. (C) Representative images of staining from the cortex and hippocampus of AD groups (*n* = 4 mice per group, three tissue sections per mouse). All images were taken at ×4 magnification on the same level of exposure and imported into ImageJ in TIFF format for quantification while the inset image was taken at ×40 magnification. Scale bars represent 200 µm. (D) No differences in the relative number of amyloid plaques could be found in the cortex across the groups. (E) Although AD mice treated with curcumin showed a reduced %area fraction of plaques relative to age‐matched vehicle controls, the difference was not significant (*p* = 0.1137). (F) The relative number of amyloid plaques also remained unchanged in the hippocampus across the groups. (G) No differences in the %area fraction of amyloid plaques could be detected in the hippocampus. Data presented as mean ± SEM. AD, Alzheimer's disease; WT, wildtype. [Color figure can be viewed at wileyonlinelibrary.com]

Brain sections were stained with Congo red to determine if curcumin reduces the number and size of amyloid plaques. Excessive amyloid plaques are a major pathology in the brain of people with AD.^32^ This pathology is recapitulated in AD mice, which start developing the plaques as early as 6 months.^24^ Congo red is a dye that binds and labels amyloids derived from various protein species in human tissue.^33^ It has widely been used to label and quantify amyloid plaques in AD mice, suggesting that it was appropriate for this study.^25^, ^26^, ^34^ Diffuse fluorescent particles were found in the cortex of AD mice aged 9 months. The absence of these particles in WT controls of the same age confirmed that they were amyloid plaques (Figure 2A,B). Figure 2C shows representative images from each of the three groups used in the statistical analysis. An outline of each brain region, the cortex and hippocampus, is also shown to illustrate the areas used in the analysis. No differences in the number of plaques in the cortex were detected across the groups, including between the baseline AD and vehicle groups (Figure 2D). Although the vehicle group showed a higher %area fraction of plaques in the cortex, the difference was not significant (Figure 2E). For the hippocampus, no differences were found across the groups for the number of plaques (Figure 2F). Consistent with the cortex, the %area fraction was increased in the vehicle group relative to AD (9 months), but not significantly (Figure 2G). These results show that curcumin had no effect on the size or number of amyloid plaques in the cortex or hippocampus.

### 3.2 Curcumin had no effect on the number or size of Aβ‐positive plaques

**Figure 3:**
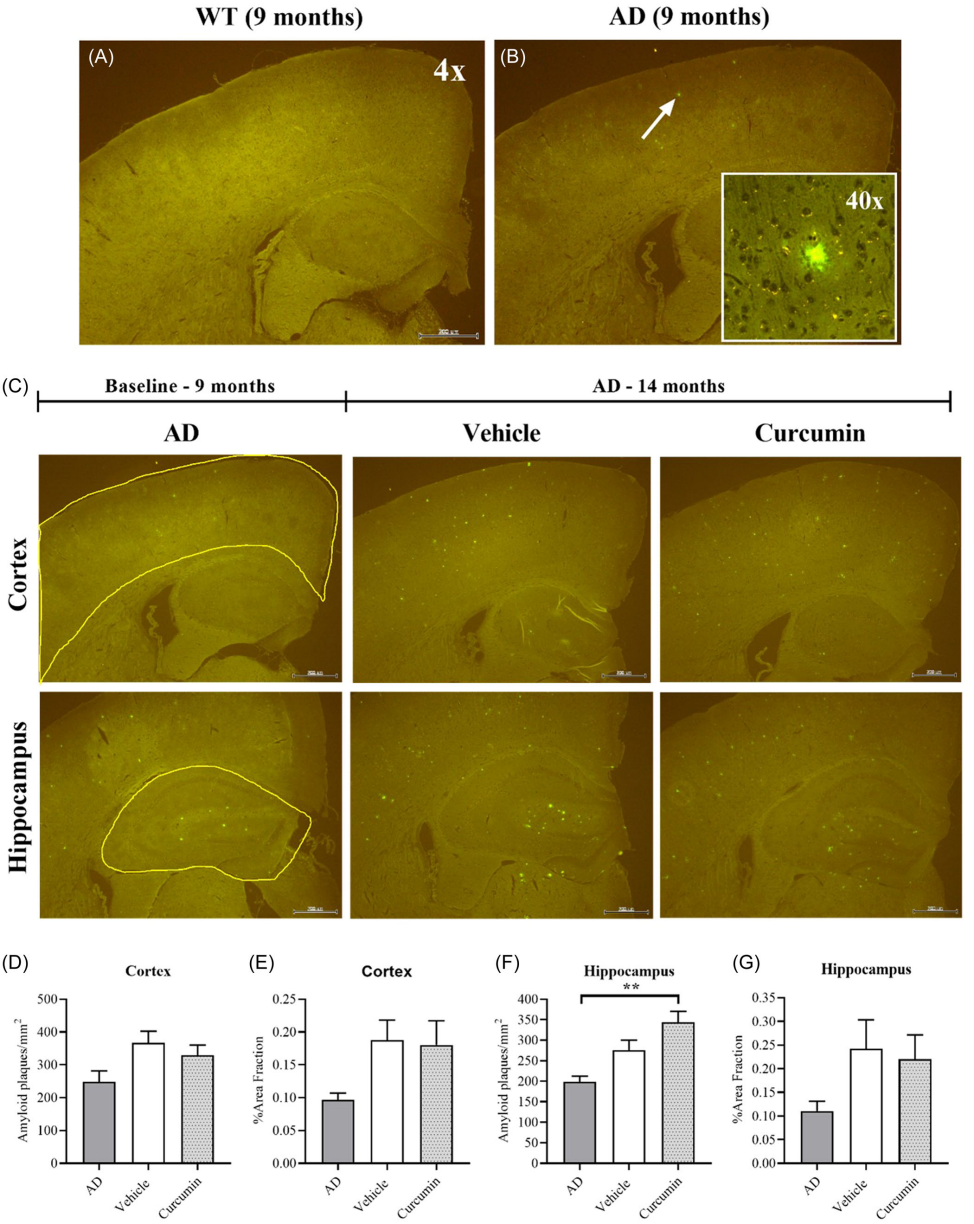
Detecting Aβ‐positive plaques in the cortex and hippocampus of AD mice using 6E10 immunohistochemistry. (A, B) Fluorescent particles were detected in the cortex of AD mice at 9 months of age (arrow in the main figure), which appeared diffuse under higher magnification (inset). These particles were absent in WT mice of the same age confirming the presence of Aβ‐positive amyloid plaques in the disease model. (C) Representative images of brain sections containing the cortex and hippocampus from the baseline AD group and vehicle and curcumin‐treated mice aged 14 months (*n* = 4 mice per group, three tissues per mouse). Images were imported into ImageJ and quantified using the same methods as for Congo red. Scale bars represent 200 µm. (D) No differences in the number or size of Aβ‐positive amyloid plaques in the cortex could be detected across the groups. (E) Although the size of the plaques appeared larger in the vehicle group relative to AD (9 months), the difference was not significant (*p* = 0.1672). (F) A significant increase in the number of Aβ‐positive amyloid plaques was detected in the hippocampus for the curcumin group (14 months) relative to AD (9 months) (*p* = 0.0036). (G) No differences in the size of Aβ‐positive amyloid plaques were detected across the groups in the hippocampus. Data presented as mean ± SEM, **p < 0.01. AD, Alzheimer's disease; WT, wildtype. [Color figure can be viewed at wileyonlinelibrary.com]

To determine if curcumin reduces the number and size of Aβ‐positive amyloid plaques, 6E10 immunohistochemistry was used. Congo red is insufficient for the assessment of amyloid plaques as it lacks specificity and can give false‐positive results.^33^ For this reason, it is often supplemented with 6E10 immunohistochemistry, which labels amyloid plaques specific to AD.^25^, ^26^ 6E10 is an antibody that recognizes the N‐terminal regions of Aβ_1–40_ and Aβ_1–42_ peptides but may also react with breakdown products of Aβ.^29^ Staining of brain sections from AD mice (9 months) with 6E10 revealed fluorescent particles similar in size to those detected by Congo red. The absence of these particles in WT mice of the same age demonstrated that they were Aβ‐positive (Figure 3A,B). Representative images of tissue sections from each AD group along with an outline of the brain regions included in the analysis are shown in Figure 3C. No differences in the number of Aβ‐positive plaques could be found in the cortex across the groups (Figure 3D). Although the %area fraction was increased in the vehicle group relative to AD (9 months), the difference was not significant (Figure 3E). In the hippocampus, an increase in the number of Aβ‐positive plaques was found for the curcumin group relative to AD (9 months), but not vehicle (Figure 3F). The %area fraction was increased in the vehicle (14 months) relative to AD (9 months), but not significantly (Figure 3G). Consistent with Congo red, these results show that curcumin had no effect on the size or number of amyloid plaques in the brain.

### 3.3 Mechanisms that target APP to the amyloidogenic pathway were not altered by curcumin

**Figure 4:**
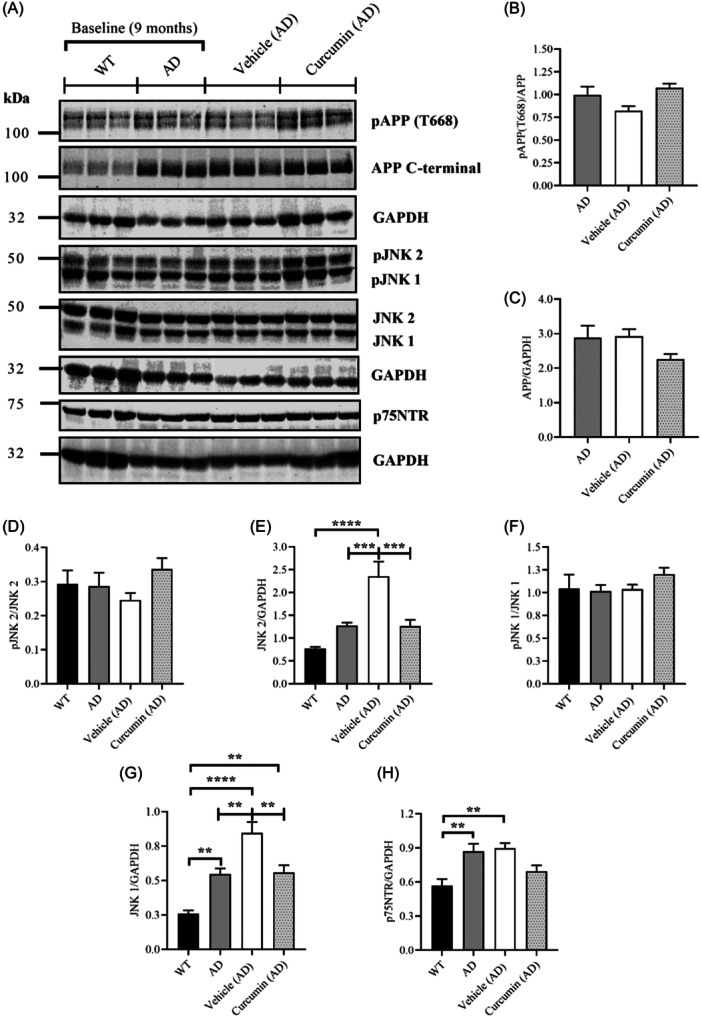
Measuring signaling pathways that indirectly regulate the production of Aβ. (A) Representative immunoblots for phosphorylated amyloid precursor protein (APP) at Threonine 668, pAPP (T668), APP C‐terminal (total APP), phosphorylated JNK (pJNK), and p75NTR along with internal loading controls. Full blots consisted of 6 samples per group. (B) Only the AD groups were included in the analysis of pAPP (T668) as WT mice did not express sufficient total APP to make a meaningful comparison (refer to blots). Although the curcumin group showed an increase in pAPP (T668) relative to the vehicle, the difference was not significant (*p* = 0.0524). (C) Total APP levels (APP C‐terminal) remained unchanged across the groups. (D, F) To confirm the results for pAPP (T668), the activity of one of the kinases that target the residue, cJun‐N‐terminal kinase (JNK), was measured. Phosphorylation of both JNK isoforms, JNK 1 and 2, were unchanged across the groups. (E) However, total protein levels for JNK 2 were significantly increased for the vehicle group relative to WT and AD baseline controls (*p* < 0.0001 and *p* = 0.0009, respectively). This increase was significantly reduced by curcumin (*p* = 0.0009). (G) Total protein levels for JNK 1 were also significantly increased for the vehicle group relative to WT and AD baseline controls (*p* < 0.0001 and *p* = 0.0040, respectively). A significant increase in the AD group relative to WT was also found (*p* = 0.0057). The increased levels of total JNK 1 in the vehicle group were significantly reduced by curcumin (*p* = 0.0057). (H) p75NTR was also measured to further explore the effects of curcumin on pathways that indirectly regulate Aβ production. p75NTR was significantly increased in the AD group relative to WT (*p* = 0.0079) and vehicle relative to WT (*p* = 0.0037). No other differences were detected. Data presented as mean ± SEM, **p < 0.01, ***p < 0.001, ****p < 0.0001. AD, Alzheimer's disease; WT, wildtype.

To determine if curcumin modulates Aβ production, signaling pathways that direct APP to the amyloidogenic pathway were measured (Figure 4A–H). Aβ is generated following sequential cleavage of APP by β‐site α‐converting enzyme 1 (BACE1) and γ‐secretase, respectively.^35^ Since BACE1 is most active at low pH, Aβ production depends on the convergence of BACE1 with APP in late endosomes/lysosomes.^36^ Phosphorylation of APP at Threonine 668, pAPP (T668) targets APP to the amyloidogenic pathway.^37^ It is also increased in post‐mortem brain tissue from people with AD, suggesting that it is aberrantly phosphorylated in the disease.^6^ pAPP (T668) was measured in this study to determine if curcumin had any effect on mechanisms that target APP to the amyloidogenic pathway. However, no differences in pAPP (T668) could be found in the brains of AD mice across the groups (Figure 4B). Relative levels of total APP protein were also unchanged (Figure 4C). The effect of curcumin on the activity of cJun‐N‐terminal kinase (JNK) was also measured. JNK targets APP at T668, suggesting that it may be one of the kinases implicated in its abnormal phosphorylation.^8^ Two bands were detected for JNK, the upper corresponding to JNK 2 (p54 isoform) and the lower JNK 1 (p46 isoform).^38^ No differences in phosphorylation of either JNK isoform were detected across the groups (Figure 4D,F). However, expression of both JNK isoforms was significantly increased in the vehicle group (aged 14 months) relative to WT and AD baseline controls (aged 9 months) (Figure 4E,G). These increases in total protein levels were significantly reduced by curcumin (Figure 4E,G). Nevertheless, the lack of changes in pJNK was consistent with the findings for pAPP (T668). Taken together, these results suggest that curcumin had no effect on signaling pathways that target APP to the amyloidogenic pathway.

p75NTR was measured to further investigate the effects of curcumin on mechanisms that target APP to the amyloidogenic pathway. p75NTR promotes the co‐localization of APP with BACE1 and its subsequent internalization into early endosomes.^8^ An increase in p75NTR, therefore, may reflect an increase in signaling pathways that facilitate Aβ production. A significant increase in p75NTR was detected in the vehicle group (aged 14 months) relative to WT and AD baseline controls (age 9 months) (Figure 4H). Although p75NTR was reduced in the curcumin group, the reduction was not significant.

### 3.4 Mechanisms that regulate the localization of BACE1 were not altered by curcumin, but TACE activity was reduced

**Figure 5:**
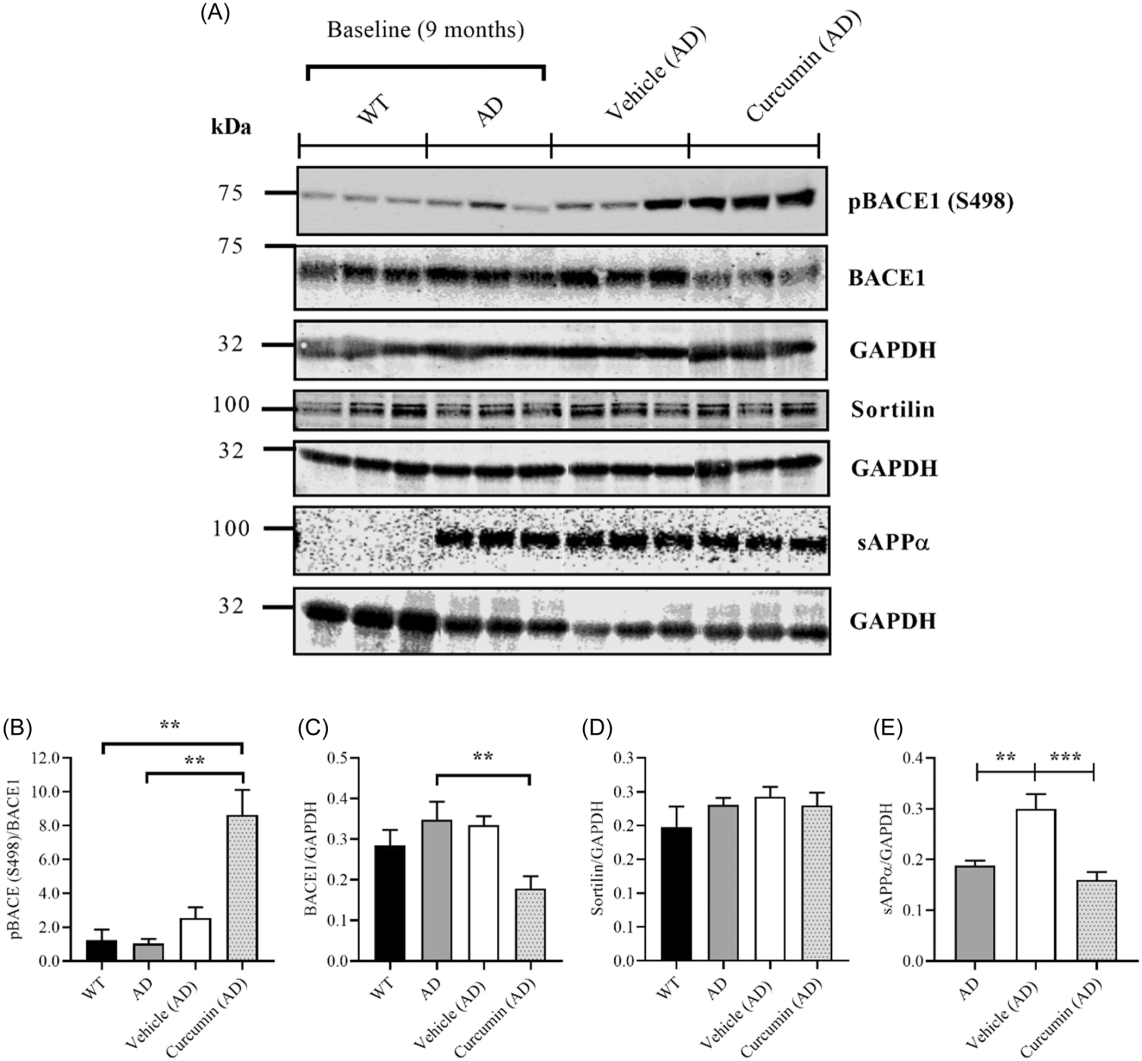
Measuring phosphorylation of BACE1 in the C‐terminal domain and tumor necrosis factor converting enzyme (TACE) activity. (A) Representative blots for pBACE1 (S498), total BACE1, sortilin, and soluble APPα (sAPPα) along with internal loading controls. Full blots consisted of six samples per group. (B) pBACE1 (S498) was significantly increased in the curcumin group relative to WT and AD baseline controls (*p* = 0.0013 and *p* = 0.0070, respectively). Although curcumin increased pBACE1 (S498) relative to vehicle more than threefold, the difference was not significant (*p* = 0.3613). (C) Total protein levels of BACE1 were significantly reduced for the curcumin group relative to AD baseline control (*p* = 0.0237). (D) Sortilin was measured as it may facilitate amyloidogenesis by binding to BACE1 and targeting it to the endolysosomal pathway. No changes in sortilin were found across the groups. (E) Protein levels of the N‐terminal fragment produced by TACE cleavage of amyloid precursor protein (APP), sAPPα, were used to determine the enzyme activity. Increased TACE activity could suggest reduced amyloidogenic processing of APP by BACE1. sAPPα was significantly increased in the vehicle group relative to AD baseline control (*p* = 0.0029). This increase was significantly reduced by curcumin (*p* = 0.0004). Data presented as mean ± SEM, **p < 0.01, ***p < 0.001. AD, Alzheimer's disease; WT, wildtype.

To further investigate the effects of curcumin on Aβ production, phosphorylation of BACE1 was measured (Figure 5A–E). Serine 498 (S498) is a residue located within a sorting motif of the BACE1 C‐terminal domain.^39^ Phosphorylation of this residue promotes BACE1 recycling from early endosomes back to the cell surface^39^ and its retrograde trafficking back to the trans‐golgi network (TGN).^40^ Therefore, pBACE1 (S498) may attenuate Aβ production by diverting BACE1 away from late endosomes/lysosomes. pBACE1 (S498) was significantly increased in the curcumin group relative to WT and AD baseline controls, but not vehicle (Figure 5B). Total protein levels of BACE1 were also significantly reduced in the curcumin group relative to AD baseline controls (Figure 5C). To further investigate the effects of curcumin on the intracellular trafficking of BACE1, levels of sortilin were measured. Sortilin is a type 1 transmembrane receptor that may facilitate amyloidogenesis by binding to BACE1 and targeting it to the lysosomal pathway.^41^ The receptor is increased in brain homogenate from people with AD and APP/PS1 mice, suggesting that its expression is deregulated in the disease.^42^ No changes in the expression of sortilin could be found across the groups in this study (Figure 5D).

To determine the effects of curcumin on non‐amyloidogenic processing of APP, tumor necrosis factor converting enzyme (TACE) activity was measured. In the non‐amyloidogenic pathway, TACE cleaves APP at the cell surface within the Aβ domain to yield an N‐terminal fragment, soluble APPα (sAPPα).^41^ The membrane‐anchored C‐terminal fragment is then further processed to yield the P3 peptide, which is not prone to aggregation. An increase in TACE activity could suggest decreased amyloidogenic processing of APP and less production of peptides prone to aggregation. A significant increase in sAPPα was detected in the vehicle group relative to AD baseline control (Figure 5E). This increase was significantly reduced by curcumin, suggesting that curcumin reduced TACE activity (Figure 5E).

### 3.5 Curcumin had no effect on the number of reactive astrocytes in the hippocampus, but increased GFAP protein levels overall

**Figure 6:**
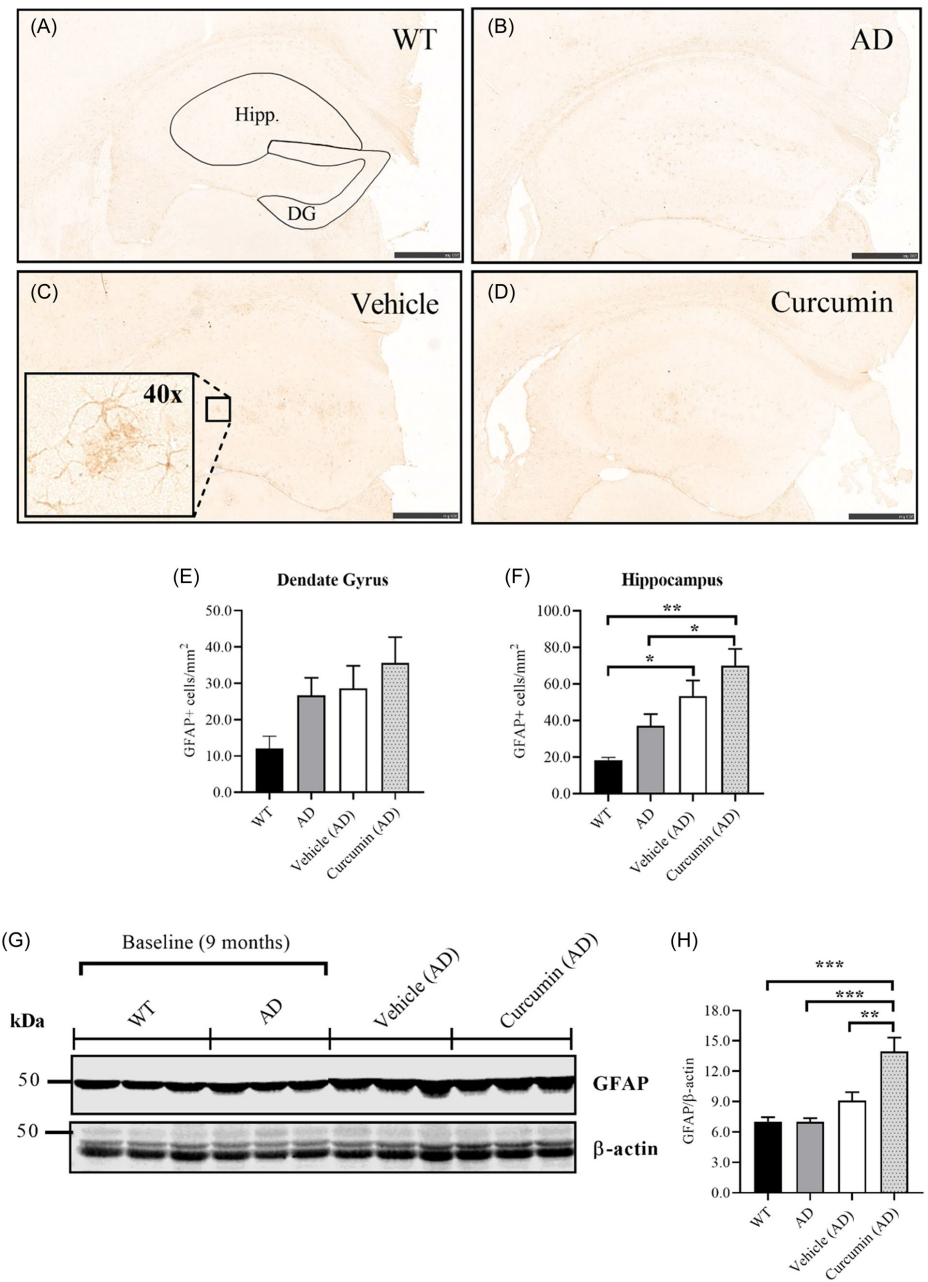
Measuring astrocyte reactivity in the hippocampus and GFAP protein levels in brain homogenate. (A–D) Representative images of coronal sections from each of the groups stained with GFAP. (E) No significant differences could be detected across the groups in the dentate gyrus. (F) The relative number of GFAP‐positive astrocytes was significantly increased in the hippocampus of vehicle and curcumin‐treated AD mice relative to WT (*p* = 0.0252 and *p* = 0.001, respectively, *n* = 4 mice per group, except vehicle—*n* = 3; minimum of two tissues per mouse). A significant increase was also found for the curcumin group relative to AD (aged 9 months) (*p* = 0.0240, *n* = 4 mice per group, except vehicle—*n* = 3; minimum of two tissues per mouse). Main images taken at ×5 magnification, inset image taken at ×40 magnification. Scale bars in main images = 500 µm. (G) To further investigate astrocyte reactivity, GFAP was measured in brain homogenate with western blot analysis. (H) Relative amounts of GFAP were significantly increased in the curcumin group relative to vehicle, AD and WT groups (*p* = 0.0065, *p* = 0.0002 and *p* = 0.0001 respectively, *n* = 6 samples per group). Data presented as mean ± SEM, *p < 0.05, **p < 0.01, ***p < 0.001. AD, Alzheimer's disease; DG, dentate gyrus; GFAP, glial fibrillary acidic protein; Hipp, hippocampus; WT, wildtype. [Color figure can be viewed at wileyonlinelibrary.com]

To determine if curcumin attenuates astrocyte reactivity, GFAP was measured in the hippocampus. GFAP is an intermediate filament protein expressed by astrocytes that have become reactive in response to neuropathology.^43^ Astrocytes positive for GFAP surround amyloid plaques in AD and release chemokine ligands that attract microglia to the site.^11^ This response worsens as the disease progresses, suggesting that it may exacerbate the neurodegeneration initiated by amyloid plaques.^12^ To determine if curcumin slows the progression of astrocyte reactivity, GFAP was stained in brain sections containing the hippocampus (Figure 6A–D). No differences in the number of GFAP‐positive astrocytes could be detected across the groups in the dentate gyrus (Figure 6E). Although they were significantly increased in the hippocampus of curcumin‐treated mice relative to WT and AD controls, they were not significantly changed relative to vehicle (Figure 6F). These results suggest that curcumin had no effect on the number of reactive astrocytes in the hippocampus.

To confirm the findings from immunohistochemistry, GFAP protein levels were measured in brain homogenate. The previous analysis of astrocyte reactivity was confined to the hippocampus. To expand the analysis to the whole brain, GFAP protein levels were measured in brain homogenate with western blot analysis. GFAP protein levels were significantly increased in the curcumin‐treated group relative to all other groups (Figure 6G–H). No other differences between groups were found. Taken together, the results indicate that curcumin did not alter the number of reactive astrocytes in the hippocampus but increased overall GFAP protein levels in the brain.

## 4 DISCUSSION

This study has evaluated the efficacy of a bioavailable curcumin formulation for the treatment of AD in mice that model the disease. The formulation was previously optimized to improve curcumin's solubility and stability in drinking water to increase its clinical utility.^21^ Although it was previously shown to improve learning and memory in AD mice, a neuropathological assessment had not been performed.^23^ The purpose of this study was to expand the preclinical evaluation by determining its effects on AD pathologies. Data from the histological analyses showed that curcumin did not reduce the number or size of amyloid plaques. Mechanisms that regulate Aβ production were also unchanged, suggesting that the course of AD was not altered. Although the number of reactive astrocytes in the hippocampus was not altered by curcumin, GFAP protein levels were increased in total brain homogenate. This finding contrasted with previous studies and suggested that it aggravated neuroinflammation.^13^, ^14^ It is possible that the dosage used in this study was too low to achieve the desired biological effects. Previous studies that demonstrate efficacy for bioavailable curcumin in AD mice used higher doses.^23^, ^44^ Therefore, despite the enhanced bioavailability of novel curcumin formulations, moderate doses may still be required to alter the course of AD.

The lack of changes in amyloid plaques for AD mice treated with curcumin suggests that it did not reduce plaque burden in the brain. Analysis of brain sections stained with Congo red failed to reveal any differences in the number or size of amyloid plaques in AD mice treated with curcumin. This result was consistent with 6E10 immunohistochemistry, suggesting that curcumin did not clear plaques from the brain. These negative results contrast with the anti‐amyloidogenic effects of curcumin that have previously been demonstrated in AD mice.^13^, ^16^ A diet supplemented with standard curcumin was shown to reduce plaque burden in the hippocampus,^16^ while standard curcumin in drinking water reduced plaque burden in the prefrontal cortex (PFC) and dentate gyrus.^13^ Greater reductions were observed with a bioavailable formulation in some brain regions,^13^ suggesting that these effects could be dose‐dependent. However, comparisons between this study and others are limited to plaque number and size, which is only one aspect of amyloid pathology.^33^ Previous studies showed that curcumin disaggregated Aβ oligomers and prevented their formation in addition to reducing plaque number and size.^13^, ^16^ Since this aspect of amyloid pathology was not measured, it remains unknown if the effects of curcumin are consistent with previous studies.

Curcumin had no effect on mechanisms that target APP to the amyloidogenic pathway, suggesting that it did not attenuate amyloidogenesis. No changes in pAPP (T668), which regulates Aβ production,^37^ could be found in the brain of AD mice treated with curcumin. This finding was consistent with the lack of changes in JNK activity, a known APP kinase,^8^ suggesting that curcumin did not attenuate APP phosphorylation. Several studies suggest that APP and tau hyperphosphorylation, which are pathological features of AD, result from an imbalance between kinase and phosphatase activity.^37^, ^45^ The modulation of these enzymes by curcumin, therefore, might reflect its potential to alter the pathophysiology of AD. Curcumin reverses Aβ_42_‐induced impairments in phosphatidylinositol 3‐kinase (PI3K)/protein kinase B (Akt) signaling, a pathway that suppresses glycogen synthase kinase‐3beta (GSK‐3β) activity, in rat models of AD.^20^, ^46^ GSK‐3β targets APP (T668) and tau,^47^ so it seems that curcumin may indirectly attenuate APP and tau hyperphosphorylation. However, in the studies, the restorative potential of curcumin was investigated after perturbations in PI3K/Akt signaling had been established.^20^, ^46^ Since JNK activity was not increased in AD mice in this study, curcumin may not have exerted any effect because there were no perturbations to restore. Nevertheless, p75NTR was increased in the brain of AD mice and curcumin did not attenuate this increase. p75NTR is an intermediate in a cycle between extracellular Aβ_42_ and JNK‐mediated pAPP (T668), suggesting that its increased expression aggravates amyloidogenesis.^8^ The lack of changes in p75NTR following treatment with curcumin suggests that it did not attenuate mechanisms that target APP to the amyloidogenic pathway.

The effects of curcumin on enzymes that process APP remain unclear, as conflicting data were obtained for BACE1 and TACE activity. Although BACE1 was reduced in AD mice treated with curcumin, the reduction was only significant compared to the baseline control, and not vehicle. This reduction could not be attributed to the effects of curcumin as the mice were not age‐matched. Nevertheless, the result is consistent with an in vitro study which showed that curcumin reduces BACE1 mRNA and protein levels in a dose‐dependent manner.^48^ Since curcumin was administered at a relatively low dose in this study, a higher dose may have provided a greater reduction. The effects of curcumin on signaling mechanisms that regulate BACE1 trafficking conflicted with the results for TACE activity. Curcumin caused an approximately threefold increase in pBACE1 (S498) relative to vehicle, which, though not significant, was still substantial. pBACE1 (S498) increases BACE1 recycling from early endosomes back to the cell surface^39^ and retrograde trafficking back to the TGN.^40^ Since BACE1 is most active in endosomal/lysosomal compartments,^36^ an increase in pBACE1 (S498) should reflect reduced amyloidogenic processing. However, curcumin also significantly reduced sAPPα, the cleavage product of TACE,^35^ suggesting that it reduced non‐amyloidogenic processing. It seems unlikely that curcumin would simultaneously reduce BACE1 and TACE activity since APP must be degraded somehow. Consequently, the analysis in this study could not determine the effects of curcumin on APP processing.

The increased levels of GFAP found in the brain of AD mice treated with curcumin suggest that it aggravated neuroinflammation. Staining of brain sections with GFAP revealed that curcumin had no effect on the number of reactive astrocytes in the dentate gyrus of AD mice. Although an increase was found in the hippocampus, the difference was only significant relative to the AD control, and not vehicle, so the effect could not be attributed to curcumin. Analysis of GFAP in total brain homogenate, however, revealed that curcumin caused an increase in overall levels of the protein. Together with immunohistochemistry, this finding suggests that curcumin did not reduce the number of reactive astrocytes but did increase the expression of GFAP. These results contrast with the effects of curcumin that have previously been demonstrated in AD mice.^13^, ^14^ Reductions in the number of GFAP‐positive astrocytes were reported after acute administration of standard and bioavailable curcumin in various brain regions of 5xFAD mice.^13^ This reduction corresponded with a reduced number of ionized calcium‐binding adaptor molecule 1 positive (Iba‐1+) microglia and an attenuation of their morphological complexity. Iba‐1 is a cytoplasmic protein involved in cytoskeletal re‐organization that is upregulated during increased membrane ruffling and cell motility.^49^ Similar reductions in GFAP protein levels have also been observed in p25‐inducible transgenic mice, which display hallmarks of AD.^14^ As reactive astrocytes secrete chemokine ligands that attract microglia to plaques,^11^ reductions in GFAP in response to curcumin would suggest reduced inflammation. Consequently, the increased levels of GFAP in this study suggest that curcumin aggravated neuroinflammation. Whether this effect was protective or destructive is uncertain as responses by astrocytes are not always damaging.

Amyloid and tau trigger differential responses in astrocytes, some of which are neuroprotective and others neurodegenerative.^50^ Postmortem analysis of human AD brains shows a positive correlation between plaque‐associated GFAP‐positive astrocytes, Iba‐1‐positive microglia, and symptom duration.^12^ This finding suggests that the inflammatory response has a damaging effect on the brain over the course of the disease.^12^ However, recent evidence from AD transgenic mice suggests that some reactions by astrocytes may be protective. At 12 months, the translation of genes that encode components of the lysosomal, apoptotic, inflammatory, and proteolytic pathways are induced above levels of WT.^50^ This finding indicates that the capacity of astrocytes to clear amyloid plaques is increased, which is neuroprotective.^50^ However, genes that encode components of the oxidative phosphorylation pathway and ribosomal components are repressed, suggesting that metabolism is impaired.^50^ These differential responses indicate that some astrocytes acquire protective functions while others acquire detrimental ones. Consequently, while GFAP may reflect astrocyte activation, it may not reflect the effect of this activation on the brain. In conclusion, it remains uncertain whether the increased levels of GFAP in response to curcumin had a protective or detrimental effect.

The neuropathological assessment in this study was mostly restricted to amyloid pathology, which limited the evaluation. The APPswe/PS1dE9 double transgenic mice used in this study have mutations in genes linked to familial AD which cause the overproduction of Aβ.^24^ Extensive amyloid plaques are apparent throughout the cortex by 6 months of age, so the mice reflect amyloid pathology.^51^ However, APPswe/PS1dE9 mice also develop many other pathologies that are characteristic of AD.^51^ Deficits in synapse number and structure,^15^ reduced expression of plasticity‐related genes,^52^ and reductions in post‐synaptic proteins ^53^ have been identified in the hippocampus. In addition, the mice also develop intraneuronal tau inclusions, but only at later stages (> 18 months).^51^ An analysis of synaptic proteins in addition to behavioral testing could have provided a more complete evaluation of the efficacy of the curcumin formulation.

## 5 CONCLUSION

Future studies on the efficacy of curcumin in AD mice could benefit from the use of moderate doses and a comprehensive biochemical analysis. The negative results from this study are not necessarily a poor reflection of the clinical utility of curcumin. As discussed, bioavailable formulations have already been developed and shown to effectively counter neuropathologies in AD mice. However, the negative results do suggest that curcumin needs to be administered in moderate doses to achieve biological effects. This requirement still exists despite the enhanced oral bioavailability that has been achieved with novel formulations. A comprehensive biochemical analysis in AD mice may also be required to properly evaluate bioavailable curcumin. AD mice reflect more than just amyloid pathology, so a complete analysis of all the pathologies in these mice may be necessary. The incorporation of these insights into future preclinical studies on the efficacy of curcumin in AD mice could result in more positive outcomes. Moreover, whether the neuroinflammation aggravated by curcumin exerts an overall protective effect or a detrimental one should be further explored.

## AUTHOR CONTRIBUTIONS

Xin‐Fu Zhou and Larisa Bobrovskaya conceived the experimental design, provided supervision during the research, and proofread the article. Clive Prestidge provided supervision during the research and proofread the article. Shaun Cade performed all experimental work and statistical analyses and prepared the manuscript.

## CONFLICT OF INTEREST STATEMENT

The authors, Larisa Bobrovskaya, the Editorial Member, and Xin‐Fu Zhou, the Editor‐in‐Chief of Ibrain, were both excluded from all decisions. Editorial decision‐making is handled independently by Editor‐in‐Chief, Professor Ting‐Hua Wang to minimize bias. Xin‐Fu Zhou is a named inventor of the curcumin formulation used in this study, Chinese patent 201610267974.5. The remaining authors declare no conflicts of interest.

## ETHICS STATEMENT

All animal procedures in this study were approved by the University Animal Ethics Committee, University of South Australia (U45/19, approval date November 8, 2019).

## Data Availability

Data analyzed in this study may reasonably be requested from corresponding authors.
